# A Significant Decrease in Thermal Conductivity in Eu- and Cd-Doped ZnO Films

**DOI:** 10.3390/nano16150928

**Published:** 2026-07-28

**Authors:** Misha Khalid, Hadiqa Naaz, Ameneh Mikaeeli, Ibtasam Bin Abdul Ghani, Misbah Aslam, Ewa Przeździecka, Hafsa Mubeen, Rafał Jakieła, Aleksandra Wierzbicka, Bartłomiej Witkowski, Andreas D. Wieck, Michał Pawlak

**Affiliations:** 1 Institute of Physics, Faculty of Physics, Astronomy, and Informatics, Nicolaus Copernicus University, Grudziadzka 5, 87-100 Torun, Poland; mishakhalid@doktorant.umk.pl (M.K.); hadiqa@doktorant.umk.pl (H.N.); ameneh.mikaeeli@doktorant.umk.pl (A.M.); 2 Chair of Applied Solid-State Physics, Experimental Physics VI, Ruhr-University Bochum, Universitaetsstrasse 150, D-44780 Bochum, Germany; andreas.wieck@ruhr-uni-bochum.de; 3 Key Laboratory of Advanced Technologies of Materials, Ministry of Education, School of Materials Science and Engineering, Southwest Jiaotong University, Chengdu 610031, China; ibtasamghani@swjtu.edu.cn; 4 Department of Physics, National Center for Physics, Quaid-i-Azam University Islamabad, Islamabad 44000, Pakistan; misbahaslam217@gmail.com; 5 Institute of Physics, Polish Academy of Sciences, Al. Lotników 32/46, 02-668 Warsaw, Poland; eilczuk@ifpan.edu.pl (E.P.); mubeen@ifpan.edu.pl (H.M.); jakiela@ifpan.edu.pl (R.J.); wierzbicka@ifpan.edu.pl (A.W.); bwitkow@ifpan.edu.pl (B.W.)

**Keywords:** thermal conductivity, ZnO, doping

## Abstract

We demonstrate that dopant inhomogeneity strongly suppresses thermal conductivity in Cd/Eu co-doped, non-polar *a*-plane-oriented ZnO films grown on *r*-plane sapphire (Al_2_O_3_) by plasma-assisted molecular beam epitaxy. X-ray diffraction confirms the *a*-plane-oriented ZnO without detectable secondary phases. Cross-sectional scanning electron microscopy shows continuous films with well-defined interfaces, and secondary-ion mass spectrometry depth profiling identifies Cd/Eu incorporation through the film thickness and a sharp Zn/O drop at the substrate interface. Cross-plane thermal transport was measured at room temperature using frequency-domain photothermal infrared radiometry (PTR) and analyzed by fitting the complex PTR amplitude and phase with a multilayer heat-diffusion model. The extracted thermal conductivity (κ) spans ~3.7–6.3 W·m^−1^·K^−1^. The lowest κ values correlate with increased Eu-distribution inhomogeneity, consistent with enhanced phonon scattering and reduced effective cross-plane heat transport.

## 1. Introduction

Zinc oxide (ZnO) is a wide-bandgap II–VI semiconductor that has attracted significant research interest due to its excellent thermal and chemical stability, high electron mobility, large exciton binding energy (~60 meV), optical transparency, and environmental compatibility [1,2,3]. These intrinsic properties make ZnO a versatile platform for a range of applications, including optoelectronic devices, ultraviolet photodetectors, transparent electronics, gas sensors, and photocatalytic systems, as well as other oxide semiconductor-based technologies [4,5,6]. Beyond its well-established electronic and optical functionalities, understanding and controlling thermal transport in ZnO have become increasingly important for efficient heat management and improved device reliability [7]. Since heat conduction in ZnO is primarily governed by phonons, considerable efforts have been devoted to engineering its lattice thermal conductivity through defect engineering, strain modulation, alloying, and controlled dopant incorporation [8,9].

The thermal transport properties of ZnO are strongly influenced by its crystal structure, crystallographic orientation, and defect distribution [10]. ZnO predominantly crystallizes in the hexagonal wurtzite structure, whose anisotropic atomic arrangement results in direction-dependent thermal transport [11]. First-principles calculations have reported that the room-temperature thermal conductivity of bulk wurtzite ZnO is approximately 38.96 W m^−1^ K^−1^ along the a-axis and 59.12 W m^−1^ K^−1^ along the c-axis, corresponding to a thermal anisotropy of nearly 50%. The average thermal conductivity is approximately 51 W m^−1^ K^−1^, with representative values of *κ*_a_ ≈ 45.2 W m^−1^ K^−1^ and *κ*_c_ ≈ 62.8 W m^−1^ K^−1^ [12]. In comparison, wurtzite GaN exhibits a substantially higher thermal conductivity of approximately 329 W m^−1^ K^−1^, highlighting the relatively lower intrinsic thermal conductivity of ZnO, which originates from smaller phonon group velocities and stronger phonon anharmonicity [13].

Accordingly, epitaxial thin films provide an excellent platform for investigating phonon transport under well-controlled structural conditions [14]. In heteroepitaxial ZnO thin films grown on sapphire substrates, thermal conductivity is substantially reduced relative to bulk values due to enhanced phonon scattering at interfaces and grain boundaries [15]. Specifically, c-axis-oriented ZnO films grown on c-plane sapphire exhibit thermal conductivities of 18–24 W m^−1^ K^−1^, whereas a-axis-oriented films grown on r-plane sapphire show slightly higher values of 24–29 W m^−1^ K^−1^. These a-axis-oriented films often exhibit single-domain-like structures with relatively smooth surfaces. Their thermal conductivity decreases as film thickness decreases, indicating that phonon transport is dominant, with mean free paths ranging from ~100 nm to 1 μm [16]. This pronounced orientation dependence reflects the intrinsically anisotropic phonon dispersion of wurtzite ZnO. Because non-polar a-plane films present a different combination of in-plane and out-of-plane phonon transport directions than c-plane films, they influence the scattering and transmission of heat-carrying phonons differently at interfaces and grain boundaries [17]. Among available deposition techniques, plasma-assisted molecular beam epitaxy (PA-MBE) enables precise control over growth kinetics, crystal quality, interface sharpness, and dopant incorporation at the atomic scale [18]. In particular, non-polar a-plane (11–20) ZnO films exhibit distinct structural and thermal characteristics compared with the more extensively studied c-plane (0001) orientation, making them an excellent model system for investigating anisotropic thermal transport and dopant effects [19].

Chemical doping is an effective strategy for tailoring phonon transport and electronic properties in semiconductors [20]. Among various dopants, rare-earth elements are particularly attractive for their large atomic masses, unique electronic configurations, and ability to induce localized lattice distortions [21]. In ZnO, Eu and Cd modify the lattice and defect landscape through distinct mechanisms. Europium incorporation occurs via the formation of Eu-related oxide clusters or substitutional configurations, depending on growth conditions, and can also introduce local structural distortions and defect-related states. Europium typically incorporates as Eu^3+^, requiring charge compensation through intrinsic defects such as zinc vacancies. In contrast, cadmium substitutes isovalently and introduces lattice strain due to ionic size mismatch [22,23,24,25]. Similar rare-earth-induced modifications have been reported in other semiconductor and oxide systems, where dopant incorporation influences optical, electronic, and structural properties. Er incorporation in silicon has been reported to enable characteristic 1.54 μm emission associated with Er^3+^ intra-4f transitions, while fluorine co-doping improves emission stability by reducing thermal quenching [26]. In Ga_2_O_3_, Er doping (1–5 mol%) modifies optical and magnetic properties, while Eu^3+^ and Tb^3+^ incorporation induce lattice distortion through local structural changes [27]. Similarly, in TiO_2_, rare-earth doping at concentrations of 0.25–1 mol% modifies electronic properties by reducing the band gap and introducing impurity states associated with room-temperature ferromagnetism. Incorporation of heavier lanthanide ions is less favorable than that of lighter elements such as La, Ce, and Pr [28].

In addition to single-element doping, co-doping has emerged as an effective strategy for further modifying lattice dynamics and achieving greater suppression of lattice thermal conductivity across various material systems [29]. This enhanced reduction in lattice thermal conductivity arises from the combined and complementary phonon-scattering mechanisms introduced by multiple dopants [30,31,32]. In Bi/Sb co-doped Mg_2_Si, this approach reduces lattice thermal conductivity to 2.9 W m^−1^ K^−1^ at 753 K due to enhanced grain-boundary phonon scattering [33]. Similarly, Nb/Te co-doped WSe_2_ exhibits a significant reduction in lattice thermal conductivity, with a reported 72% decrease to 0.48 W m^−1^ K^−1^ at 850 K, attributed to enhanced phonon scattering induced by the combined effects of Nb and Te dopants [34]. Despite these advances, the synergistic effects of simultaneous Cd/Eu incorporation on phonon scattering in epitaxial, non-polar ZnO remain largely unexplored. Moreover, the role of dopant spatial distribution in governing thermal conductivity in such systems has not yet been systematically investigated.

In this work, we examine the structural, compositional, and thermal properties of non-polar a-oriented ZnO thin films co-doped in situ with trace amounts of Cd and Eu, grown on r-plane sapphire via plasma-assisted molecular beam epitaxy. Structural and morphological analyses were conducted using X-ray diffraction and scanning electron microscopy, and dopant depth profiles were obtained by secondary-ion mass spectrometry. Cross-plane thermal conductivity was evaluated using frequency-domain photothermal infrared radiometry combined with multilayer heat-diffusion modeling. The co-doped films exhibit a marked reduction in thermal conductivity compared with undoped a-plane ZnO, with lower values associated with increased defect nonuniformity. These results demonstrate that trace Cd and Eu incorporation can effectively suppress thermal transport through dopant-induced inhomogeneity and highlight the role of Eu dopant distribution in phonon scattering and thermal conductivity tuning.

## 2. Materials and Methods

In situ ZnO:Cd,Eu films were grown on commercially available *r*-plane sapphire substrates using plasma-assisted molecular beam epitaxy (PA-MBE) in a Riber Compact 21 system (Riber S.A., Bezons, France). The *r*-plane sapphire (Al_2_O_3_) substrates (CrysTec GmbH, Berlin, Germany) were chemically cleaned in a H_2_SO_4_:H_2_O_2_ (1:1) mixture for 5 min before growth. For thermal cleaning the substrates were annealed at a temperature of 150 °C for 1 h in the loading chamber and then the substrates were transferred to the growth chamber followed by vacuum annealing at 700 °C and then for 30 min in an oxygen (O_2_) plasma. During the growth process, the radiofrequency (RF) power of the oxygen plasma was maintained at 240 W with an O_2_ gas flow rate of 3 sccm. All ZnO:Cd,Eu samples were grown at 360 °C, as measured by a thermocouple. High purity Zn (6 N), Cd (6 N), and Eu (4 N) elements were used as sources from the Knudsen effusion cells. The Beam Equivalent Pressure (BEP), defined as a local pressure of a Zn and Cd and Eu directional gas beam on a substrate surface (namely fluxes), was measured by a pressure gauge. The details of the growth parameters in analyzed samples are presented in Table 1.

**Figure 1:**
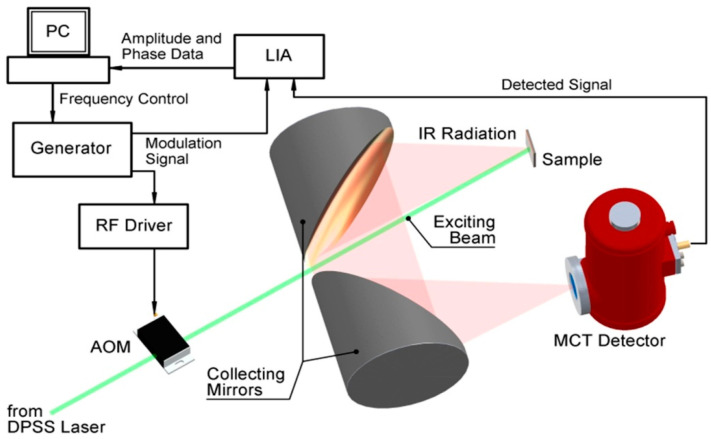
PTR experimental set-up used in the study [35].

Thermal conductivity measurements were carried out using a photothermal infrared radiometry (PTR) system schematically shown in Figure 1. A DPSS laser (CNI MGL-FN-532-1W, 532 nm, 800 mW) served as the excitation source. The laser intensity was modulated in the frequency range of 100 Hz to 2 MHz using an acousto-optic modulator (Crystal Technology 3080-120), driven by a waveform generator (Agilent 33522A). The laser beam diameter (1/e^2^) was adjusted from approximately 2.8 mm at low modulation frequencies (100–1000 Hz) to about 1.8 mm at higher frequencies to optimize the signal producing periodic thermal excitation of the sample. Infrared thermal emission from the sample was collected and collimated using two gold-coated off-axis parabolic mirrors and focused onto a liquid nitrogen cooled mercury cadmium telluride (MCT) detector (Kolmar Technologies KMPV 11-1-J1/DC/Ge), sensitive in the 2–12 µm spectral range. The detector output was analyzed using a lock-in amplifier (Stanford Research Systems SR865), providing amplitude and phase information. Data acquisition and control were performed via computer. Before measurements, the system response and one-dimensional heat flow conditions were verified using reference glass and glassy carbon samples. Under conditions where the laser beam radius significantly exceeded the thermal diffusion length, the PTR signal corresponded to one-dimensional cross-plane thermal transport [35,36].

In the case of PTR, the governing equations are expressed as follows:

(1) $$\theta_{PTR}=\;-\frac{D}{C\;}$$

*C* and *D* are defined in Equation (2) but with different value of $\sigma^{2}=\frac{1}{k_{⊥}}\left(i\omega \rho C\right)$.

(2) $$\left[A_{n}\;B_{n}\;C_{n}\;D_{n}\;\right]={\;M}_{3}{\;M}_{32}{\;M}_{2}{\;M}_{21\;}{\;M}_{1\;}$$

where *M* is defined as an interface between layers, *M*_3_ for the third layer, *M*_32_ for the interface between the third and second layer, and so on.

(3) $$A_{n}=cosh\left(\sigma_{n}l_{n}\right)=D_{n};\;B_{n}=\frac{sinh(\sigma_{n}l_{n})}{\kappa_{⊥n}\sigma_{n}};\;C_{n}=-\kappa_{⊥n}\sigma_{n}sinh\;(\sigma_{n}l_{n})$$

where $\kappa_{⊥}$ represents cross-plane thermal conductivity, α denotes thermal diffusivity and *l_n_* signifies the layer thickness of the nth layer and, ρC, signifies the volumetric specific heat.

The thermal boundary resistance at the interface, *R*, is determined by:

(4) $${\;M}_{n,n+1}=\;\left[1\;-R_{n,n+1}\;0\;1\;\right]$$

where *R*_*n*,*n*+1_ denotes the thermal interface resistance between the nth and the (*n* + 1)-th layers.

## 3. Results and Discussion

**Figure 2:**
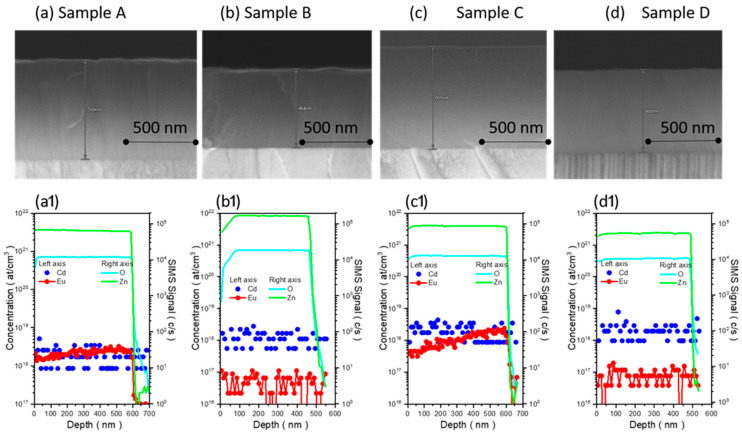
Cross-sectional SEM images (**a**–**d**) and corresponding SIMS depth profiles (**a1**–**d1**) for ZnO:Cd,Eu films: (**a**) sample A, (**b**) sample B, (**c**) sample C, and (**d**) sample D. The SEM images indicate the film thickness, while the SIMS profiles show Cd and Eu concentrations in at./cm^3^ (left axis) and Zn/O matrix signals (right axis) as a function of film depth.

A Hitachi SU-70 scanning electron microscope (SEM) (Hitachi High-Tech Corporation, Tokyo, Japan) was used to study the samples morphology. The thickness of the layers was determined from cross-section SEM measurements (Figure 2a–d). The layers obtained are continuous, and the interfaces between the ZnO:Cd,Eu layer and the sapphire substrate are well defined. The low amount of cadmium and europium doping was achieved during the growth process. In-depth Eu concentration profiles were measured on selected samples by secondary-ion mass spectrometry (shown in Figure 2(a1–d1)) but the Cd concentration was below detection limit (~10^18^ cm^−3^) and Eu close to detection limits for this equipment and method. The measurements were performed with a CAMECA IMS 6F (CAMECA, Gennevilliers, France) system. Cd- and Eu-implanted ZnO layers were used as standards.

In the case of Eu-doped ZnCdO films, the observed depth profile—lower Eu concentration near the surface and enhanced accumulation at the interface with the substrate—can be attributed to a combination of kinetic and thermodynamic effects during MBE growth. Europium atoms may exhibit limited incorporation efficiency at later growth stages, leading to gradual depletion in the regions of the layer. At the same time, Eu can diffuse toward the interface during growth or in early stages, where it becomes trapped due to a higher density of defects or strain fields. This behavior may be further enhanced by possible instabilities of the Eu effusion cell flux, which can introduce time-dependent variations in the dopant supply during growth. Additionally, changes in surface segregation and sticking coefficient as the alloy composition evolves can contribute to the formation of a non-uniform depth profile.

This interpretation is consistent with previous reports on rare-earth doping in semiconductors, where dopant segregation, diffusion, and flux instabilities during MBE growth were identified as key factors influencing depth profiles [37,38,39].

**Figure 3:**
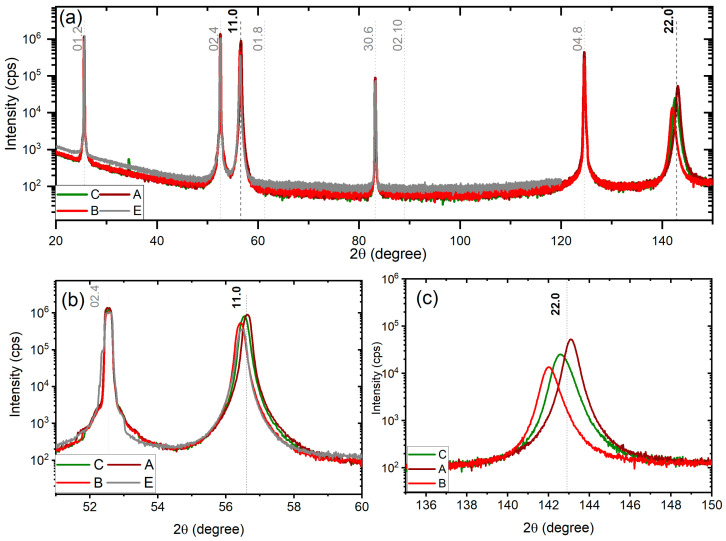
θ–2θ XRD patterns of the ZnO:Eu,Cd layers grown on r-Al_2_O_3_ (**a**) full scan; (**b**,**c**) enlarging the 110 and 220 peak areas to show their shift. The gray vertical doted lines indicate the position of Al_2_O_3_ peaks based on the JCPDS card no. 00-042-1468, whereas black doted lines indicate the position of ZnO peaks based on the JCPDS card no. 00-042-1468.

The crystalline quality of the ZnO:Eu,Cd films deposited on r-Al_2_O_3_ was assessed by X-ray diffraction measurements using a Panalytical X’Pert Pro MRD (PANalytical B.V., Almelo, The Netherlands) diffractometer equipped with a hybrid two-bounce Ge (2 2 0) monochromator and triple axis Ge (2 2 0) analyzer in front of the detector, with Cu K_α1_ radiation (λ = 1.54056 Å). Figure 3a presents the θ–2θ scans of all samples. The main peaks observed in the diffractograms are from reflections of 11.0 and 22.0 hexagonal ZnO atomic planes (JCPDS card no. 00-036-1451) and 01.2, 02.4, 30.6, 04.8 Al_2_O_3_ (substrate) atomic planes (JCPDS card no. 00-042-1468) (Figure 3a). Figure 3b,c present enlarged views of the ZnO (110) and (220) regions, respectively, highlighting systematic peak shifts relative to the reference ZnO peak positions. The MBE ZnO:Eu,Cd layers grow in *a*-direction as is expected on *r*-plane sapphire [19]. No other crystalline phases are detected, indicating a relatively high quality of the layers. The shift of diffraction peaks 11.0 and 22.0 indicates a change in the lattice constant “a”. This type of behavior can be related to the addition of both Cd and Eu to the ZnO lattice [40,41,42,43,44,45]. Table 2 presents XRD and SEM results.

**Figure 4:**
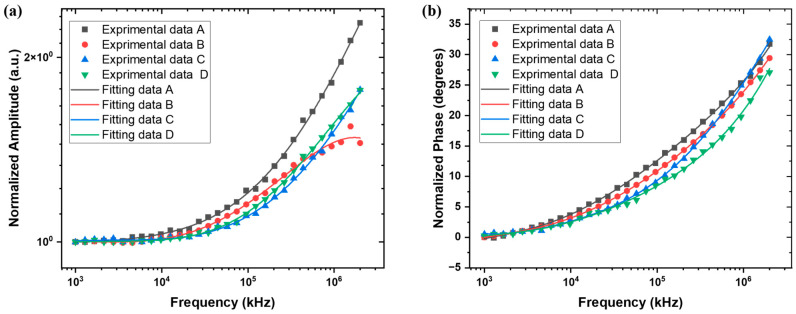
(**a**) Frequency-dependent normalized amplitude and (**b**) normalized phase with model fit of ZnO:Cd,Eu films.

Figure 4a,b show the normalized amplitude and phase vs. modulation frequency for four ZnCdO,Eu films (z1124R, z12AR, z12BR, and z13R). Experimental data are plotted as symbols, and the corresponding best-fit responses from fitting the complex PTR signal are shown as solid lines. The close overlap across the full frequency range indicates that the model accurately captures the sample thermal response, supporting the reliability of the extracted thermal parameters. From these fits, the thermal conductivity (*k*), thermal diffusivity (*α*), and thermal boundary resistance (*R*) are derived for the ZnO:Cd,Eu films, presented in Table 3.

**Figure 5:**
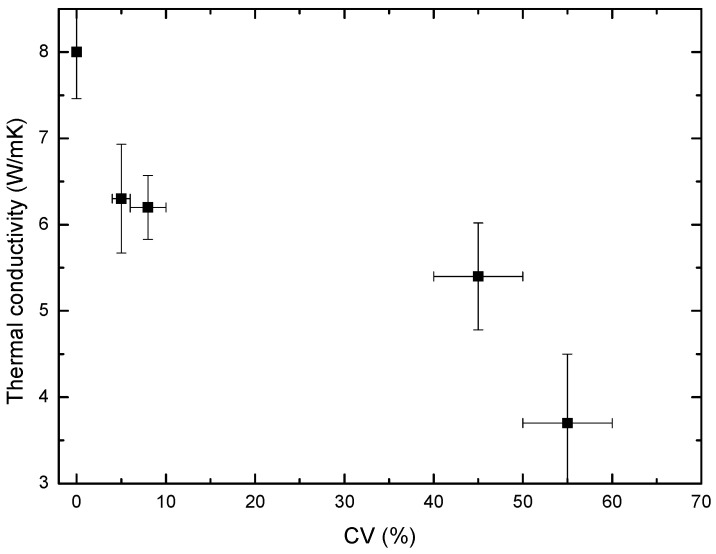
Correlation between thermal conductivity and inhomogeneity of Eu doped samples using CV parameters.

The coefficient of variation (CV) quantifies relative dispersion in SIMS depth profiles, measuring inhomogeneity in element concentrations. It is calculated as the standard deviation divided by the mean, expressed as a percentage:

(5) $$CV=\frac{\sigma }{\mu }\times 100\%$$

where *σ* is the standard deviation of the ion signal intensity (or concentration in cps/at.%) across the selected depth range, and *μ* is the arithmetic mean. Figure 5 shows the relationship between *κ* and CV.

Figure 5 shows that cross-plane thermal conductivity decreases as the heterogeneity in the Eu doping described by Equation (5). This can be explained by the fact that dopant-rich and dopant-poor regions create more interfaces, strain fields, and point-defect disorder, all of which interrupt heat-carrying phonons and reduce thermal conductivity [46].

**Figure 6:**
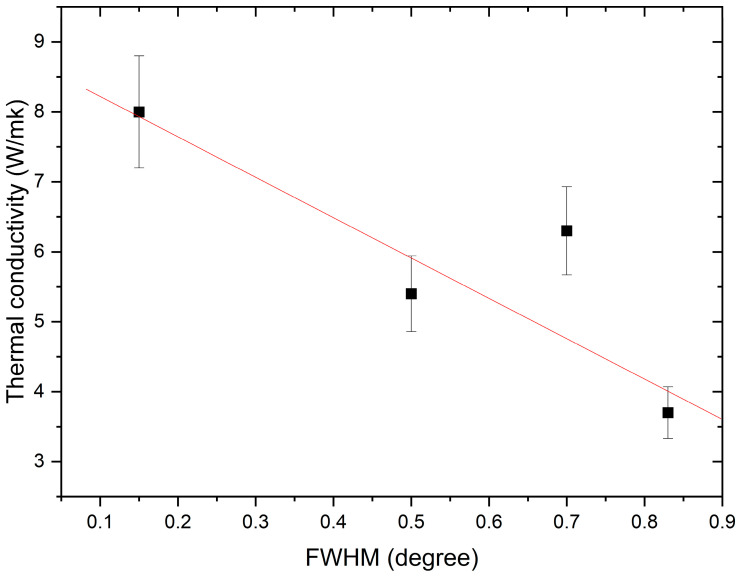
Linear correlation between cross-plane thermal conductivity and the FWHM of the (110) diffraction peak.

Dopant-induced structural disorder is expected to significantly influence phonon transport across the film thickness. To quantify this effect, the cross-plane thermal conductivity is correlated with the full width at half maximum (FWHM) broadening measured along the (110) crystallographic direction, as presented in Figure 6.

Figure 6 shows the relationship between the thermal conductivity and the full width at half maximum (FWHM) of the 11.0 ZnO diffraction peak for Cd- and Eu-doped ZnO thin films. A decreasing trend in thermal conductivity with increasing FWHM is observed. This behavior suggests that the deterioration of the crystalline quality, reflected by the broader diffraction peak, enhances phonon scattering due to a higher density of structural defects and/or smaller crystallite size, leading to reduced thermal conductivity. Structural defects act as phonon scattering centers, and phonons are the primary carriers of heat in ZnO:Cd,Eu. Consequently, an increase in FWHM leads to more intense phonon scattering and a decrease in the thermal conductivity of the studied layers.

**Figure 7:**
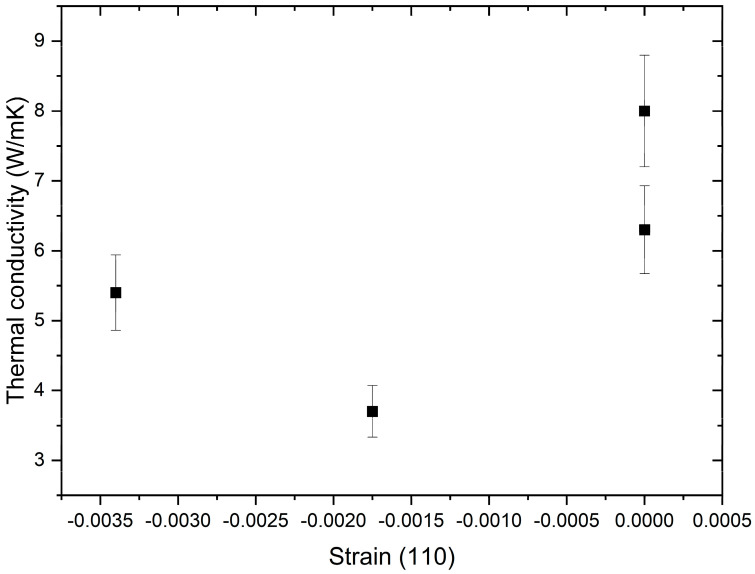
The relationship between the thermal conductivity and the residual strain determined from the 11.0 ZnO X-ray diffraction peak for Cd- and Eu-doped ZnO thin films deposited on r-plane Al_2_O_3_ substrates.

Figure 7 shows the relationship between the residual stress of Cd- and Eu-doped ZnO thin films, calculated along the film growth direction (the (110) crystallographic direction), and the thermal conductivity. The results indicate a transition from compressive to tensile residual strain with increasing thermal conductivity. The highest thermal conductivity is obtained for samples exhibiting the largest tensile strain, suggesting that tensile stress may be associated with improved crystal quality and reduced phonon scattering in Cd- and Eu-doped ZnO thin films. It should be noted that the strain analysis is restricted to a single crystallographic direction and therefore provides only partial information about the strain state in the films. This limitation arises from the fact that only standard θ–2θ X-ray diffraction measurements were carried out. The primary purpose of these measurements was to assess the crystallinity of the ZnO:Cd,Eu thin films (single-crystalline vs. polycrystalline) and to confirm the absence of secondary phases associated with Cd and Eu doping.

## 4. Conclusions

The present study demonstrates that high-quality non-polar a-oriented ZnO thin films co-doped with trace amounts of Eu and Cd can be successfully grown by plasma-assisted molecular beam epitaxy (PA-MBE). Although the Cd and Eu concentrations are close to the detection limit of the SIMS measurements, X-ray diffraction reveals measurable changes in the lattice parameters, confirming that even very low dopant concentrations modify the crystal structure. 

Cross-plane thermal conductivity measurements performed by frequency-domain photothermal infrared radiometry show a pronounced reduction in thermal conductivity compared with undoped ZnO films. The lowest thermal conductivities are observed for samples exhibiting the largest spatial inhomogeneity of the Eu distribution, indicating that dopant concentration fluctuations enhance phonon scattering and thereby suppress heat transport. Furthermore, the thermal conductivity correlates with structural parameters obtained from X-ray diffraction, including the full width at half maximum (FWHM) of the (110) diffraction peak and the residual strain, demonstrating a clear relationship between crystalline quality, defect structure, and thermal transport. 

These findings highlight that controlled incorporation of trace amounts of Eu and Cd provides an effective route for tailoring the thermal properties of epitaxial ZnO. The results contribute to a better understanding of phonon scattering mechanisms in doped oxide semiconductors and may be relevant for the design of ZnO-based materials requiring controlled heat transport. Future work should include quantitative determination of the Cd distribution, a more detailed analysis of the respective contributions of Cd and Eu to phonon scattering, and temperature-dependent thermal conductivity measurements to further elucidate the dominant heat-transport mechanisms.

## Figures and Tables

**Table 1:** Growth parameters of the oxide samples: Zn, Cd and Eu fluxes.

Sample Name	Zn Flux (×10^−7^ Torr)	Cd Flux (×10^−8^ Torr)	Eu Flux (×10^−9^ Torr)
A	9.72	4.00	1.11
B	9.72	6.00	0.05
C	9.17	3.25	0.97
D	9.85	7.20	4.73

**Table 2:** Lattice parameter a and film thickness determined by XRD and cross-sectional SEM, respectively.

Sample Name	a (Å)	Thickness (nm)
A	3.2478	589
B	3.2590	464
C	3.2527	605
D	-	488

**Table 3:** Summary of thermal conductivity (*κ*), thermal diffusivity (*α*), and thermal boundary resistance (*R*) for ZnO:Cd,Eu films and undoped ZnO reference film.

Sample Name	Thermal Conductivity (*κ*) (Wm^−1^K^−1^)	Thermal Diffusivity (α) (×10^−6^ m^2^/s)	Thermal Boundary Resistance (*R*) (×10^−7^ m^2^KW^−1^)
A (z12AR)	5.4 ± 0.5	7.2 ± 0.7	1.9 ± 0.2
B (z12BR)	6.3 ± 0.6	7.5 ± 0.7	2.6 ± 0.3
C (z13R)	3.7 ± 0.3	7.0 ± 0.6	1.6 ± 0.2
D (z1124R)	6.2 ± 0.6	7.0 ± 0.6	2.2 ± 0.2
E (ZnO)	8.0	-	-

## Data Availability

The data presented in this study are available on request from the corresponding author.
